# Vegetable nitrate intake, blood pressure and incident cardiovascular disease: Danish Diet, Cancer, and Health Study

**DOI:** 10.1007/s10654-021-00747-3

**Published:** 2021-04-21

**Authors:** Catherine P. Bondonno, Frederik Dalgaard, Lauren C. Blekkenhorst, Kevin Murray, Joshua R. Lewis, Kevin D. Croft, Cecilie Kyrø, Christian Torp-Pedersen, Gunnar Gislason, Anne Tjønneland, Kim Overvad, Nicola P. Bondonno, Jonathan M. Hodgson

**Affiliations:** 1 Institute for Nutrition Research, School of Medical and Health Sciences, Edith Cowan University, Level 3, Royal Perth Hospital Research Foundation, Rear 50 Murray St, Perth, WA 6000 Australia; 2 Medical School, The University of Western Australia, Royal Perth Hospital, Perth, WA Australia; 3 Department of Cardiology, Herlev and Gentofte University Hospital, Copenhagen, Denmark; 4 School of Population and Global Health, University of Western Australia, Perth, WA Australia; 5 Centre for Kidney Research, Sydney Medical School, School of Public Health, The University of Sydney, Sydney, Australia; 6 School of Biomedical Sciences, University of Western Australia, Royal Perth Hospital, Perth, WA Australia; 7 The Danish Cancer Society Research Center, Copenhagen, Denmark; 8 Department of Clinical Investigation and Cardiology, Nordsjælland Hospital, Hillerød, Denmark; 9 The National Institute of Public Health, University of Southern Denmark, Odense, Denmark; 10 The Danish Heart Foundation, Copenhagen, Denmark; 11 Department of Public Health, Faculty of Health and Medical Sciences, University of Copenhagen, Copenhagen, Denmark; 12 Department of Public Health, Aarhus University, Århus, Denmark; 13 Department of Cardiology, Aalborg University Hospital, Ålborg, Denmark

**Keywords:** Vegetables, Nitrate, Nitric oxide, Blood pressure, Cardiovascular disease

## Abstract

Whether the vascular effects of inorganic nitrate, observed in clinical trials, translate to a reduction in cardiovascular disease (CVD) with habitual dietary nitrate intake in prospective studies warrants investigation. We aimed to determine if vegetable nitrate, the major dietary nitrate source, is associated with lower blood pressure (BP) and lower risk of incident CVD. Among 53,150 participants of the Danish Diet, Cancer, and Health Study, without CVD at baseline, vegetable nitrate intake was assessed using a comprehensive vegetable nitrate database. Hazard ratios (HRs) were calculated using restricted cubic splines based on multivariable-adjusted Cox proportional hazards models. During 23 years of follow-up, 14,088 cases of incident CVD were recorded. Participants in the highest vegetable nitrate intake quintile (median, 141 mg/day) had 2.58 mmHg lower baseline systolic BP (95%CI − 3.12, − 2.05) and 1.38 mmHg lower diastolic BP (95%CI − 1.66, − 1.10), compared with participants in the lowest quintile. Vegetable nitrate intake was inversely associated with CVD plateauing at moderate intakes (~ 60 mg/day); this appeared to be mediated by systolic BP (21.9%). Compared to participants in the lowest intake quintile (median, 23 mg/day), a moderate vegetable nitrate intake (median, 59 mg/day) was associated with 15% lower risk of CVD [HR (95% CI) 0.85 (0.82, 0.89)]. Moderate vegetable nitrate intake was associated with 12%, 15%, 17% and 26% lower risk of ischemic heart disease, heart failure, ischemic stroke and peripheral artery disease hospitalizations respectively. Consumption of at least ~ 60 mg/day of vegetable nitrate (~ 1 cup of green leafy vegetables) may mitigate risk of CVD.

**Supplementary Information:**

The online version contains supplementary material available at 10.1007/s10654-021-00747-3.

## Introduction

Identifying evidence-based strategies to prevent cardiovascular disease (CVD) is a global research priority. An important strategy to reduce CVD risk is to identify optimal diets and their cardioprotective components. One such potentially cardioprotective component is dietary inorganic nitrate, an exogenous source of nitric oxide (NO). NO plays a key role in cardiovascular health [1]. Produced primarily through the endogenous L-arginine-NO synthase pathway, there are few options to enhance NO. The discovery that end-products of NO metabolism, nitrate and nitrite, are recycled back into NO through an enterosalivary nitrate-nitrite-NO pathway raised the possibility that dietary nitrate could be an important exogenous source of NO benefitting cardiovascular health [2]. The major dietary sources of nitrate are vegetables, particularly green leafy vegetables and beetroot [3].

Intervention studies have investigated whether inorganic nitrate, through effects on NO, improves validated markers of CVD risk [4]. This was first shown in healthy volunteers in whom significant reductions in BP (− 3.5 mmHg) were observed after ~ 400 mg nitrate intake for 3 days [5]. To date, more than 50 clinical trials (2 h to 42 day duration) have examined the effect of nitrate (sources: beetroot juice, green leafy vegetables, and nitrate salts) on BP and vascular function [4]. Meta-analyses of these studies have shown a significant inverse association between nitrate consumption and BP [6, 7] as well as beetroot juice consumption and BP [8]. However, evidence from observational studies of a reduction in CVD risk with habitual dietary nitrate intake is incomplete. Three Australian prospective studies (< 5500 participants and < 2000 incident cases each) have reported a lower risk of CVD [9–12], with higher dietary nitrate intakes while a large American prospective study (~ 60,000 participants; ~ 2300 incident cases) observed no benefit [13]. The established pathway via which dietary nitrate augments NO and accumulating evidence from clinical trials provides a strong rationale to further investigate the relationship between habitual dietary nitrate intake and risk of CVD in a large prospective study with a long follow-up, a large number of incident cases, and a comprehensive measure of nitrate intake. Furthermore, yet to be determined is the association between dietary nitrate and the individual CVD subtypes, whether a reduction in CVD risk with habitual dietary nitrate intake is mediated by effects on BP, and whether the association is different in subgroups at higher risk for CVD.

The primary aim of this study was to investigate the association between vegetable nitrate intake and incident CVD in the Danish Diet, Cancer, and Health Study cohort. Vegetable nitrate was the focus as approximately 80% of total dietary nitrate intake comes from consumption of vegetables [10, 14]. Secondary aims were to (1) investigate the association of vegetable nitrate intake with baseline systolic BP (SBP) and diastolic BP (DBP); (2) investigate whether associations with CVD are mediated by BP; and (3) investigate whether associations differ in the presence of risk factors for CVD, including sex, body mass index (BMI), smoking status, and alcohol intake.

## Methods

### Study population

Between 1993 and 1997, the Danish Diet, Cancer, and Health Study recruited 57,053 participants of the greater areas of Copenhagen and Aarhus. Of these, 56,468 completed a food-frequency questionnaire (FFQ) at baseline and were without a cancer diagnosis prior to enrolment. The following databases were cross-linked to the cohort: The Civil Registration System [15], The Integrated Database for Labor Market Research [16], The Danish National Patient Register (DNPR)[17] and The Danish National Prescription Registry [18].

Participants were excluded if they had any prevalent CVD (n = 2933), self-reported or recorded diagnosis in the DNPR. Furthermore, participants were excluded if they had missing or implausible values for any covariates (n = 192), or implausible energy intakes [< 2092 kJ/day (< 500 kcal/day) and > 20,920 kJ/day (> 5000 kcal/day), n = 193] Supplemental Figure 1.

This study was approved by the Danish Data Protection Agency (Ref No. 2012-58-0004 I-Suite nr: 6357, VD-2018-117).

### Exposures

Prior to their first visit, participants completed a 192-item validated FFQ [19]. Participants reported their average intakes of different food and beverage items over the previous 12 months.

#### Vegetable nitrate

Vegetable nitrate intake was calculated from vegetables assessed using the FFQ and quantified in grams/day. A recently published comprehensive database, with nitrate data for 178 vegetables from 255 publications, was applied to assess nitrate levels for each vegetable [3]. The nitrate content of vegetables varies according to country of cultivation; therefore, the following strategy was employed. For each vegetable, if 3 or more entries were available in the database for Northern Europe (Denmark, Estonia, Finland, Iceland, Ireland, Latvia, Lithuania, Norway, Sweden and United Kingdom), the median of these values was used. If there were less than 3 entries in the database for Northern Europe, the median of values for all European countries was used. If there were less than 3 entries available for European countries, the median of values for all countries in the database was used. Vegetable nitrate intake (mg/day) was calculated by multiplying the quantity reportedly consumed (g/day) by the median nitrate content (mg/g) of that vegetable. Based on information in the nitrate content of vegetables database [3] and nitrate loss estimates in another cohort [20], for those vegetables not consumed raw, a 50% reduction in the assigned nitrate value was applied to take into account the effect of cooking. The nitrate values from each individual vegetable were added to obtain the sum of daily vegetable nitrate values.

#### Non-vegetable dietary nitrate

Nitrate intake from non-vegetable items, excluding water, was calculated using values from Inoue-Choi et al. [21]. Nitrate intake was calculated by multiplying the reported quantity of consumption for each food item (g/day) by its assigned mean nitrate value (mg/g). Total non-vegetable nitrate intake (mg/day) was determined by calculating the sum of nitrate values from all items included in the FFQ, excluding vegetables.

### Study outcomes

#### Blood pressure

Baseline SBP and DBP was measured by trained staff members using automated oscillometric sphygmomanometers (model UA 751 or UA-743; Takeda Pharmaceutical Co. Ltd., Osaka, Japan). A measurement was taken on the right arm, with participants lying in a supine position after at least 5 min rest and a minimum of 30 min after tobacco smoking and intake of food, tea, or coffee. Where a participants’ SBP was ≥ 160 mmHg, or DBP was ≥ 95 mmHg, the measurement was repeated after 3 min, with the lower of the two measurements used.

#### Incident CVD and CVD subtypes

For the time-to-event analysis, the primary outcome was a combined endpoint of first-time incident CVD. Incident CVD was defined as a hospitalization with a primary or secondary diagnosis of ischemic heart disease (IHD), ischemic stroke, hemorrhagic stroke, peripheral artery disease (PAD), heart failure, or inpatient or outpatient diagnosis of atrial fibrillation (AF). Secondary outcomes were: IHD, ischemic stroke, hemorrhagic stroke, PAD, heart failure, and AF discretely. All outcomes were identified by ICD-10 codes using the DNPR. These ICD codes have previously been validated for research purposes in the DNPR with positive predictive values (PPVs) of 92–97% for IHD [22], 81 – 85% for ischemic stroke [23, 24], 88% for hemorrhagic stroke [25], 81% for PAD [26], 76% for heart failure [22] and 95% for AF [22]. We did not include a diagnosis of unstable angina or transient ischemic attack as these in the DNPR lack validity for research purposes.

### Validated case analysis

Using only medically reviewed and validated cases of certain CVD diagnoses [myocardial infarction (MI), ischemic stroke, and PAD] during follow-up, we investigated associations to verify our results based on the ICD-10 codes for outcomes. Patients with an ICD-10 discharge code for ischemic stroke, PAD or MI up until 2009, registered as a primary or secondary diagnosis, were considered possible cases to be reviewed (Supplemental Table 1). Due to a prior diagnosis of validated CVD, a further seven participants were excluded in this analysis (n remaining in analysis = 53,143). The methods of case validation have been published previously [24, 26, 27].

### Covariates

Information on age, sex, education, smoking habits, alcohol consumption, and daily activity was obtained from self-administered lifestyle questionnaires completed by participants. Dietary data were obtained from the semi-quantitative FFQ described above. Anthropometric measurements were taken, and SBP, DBP, and cholesterol levels were measured at the study centers. Household annual income after taxation and interest for the value of the Danish currency was averaged over the 5-years immediately prior to study enrolment in 2015. ICD-8 and ICD-10 codes were used for diagnosis of chronic kidney disease, chronic obstructive pulmonary disease, and cancers. For diabetes mellitus, only self-reported data were used due to the low validity of ICD-codes in DNPR [28]. For treatment of diabetes mellitus, both self-reported data and data on filled prescription for insulin and non-insulin medication were used. Use of antihypertensive medication was obtained from self-reported use or the use of two or more antihypertensive medications within 180 days prior to study enrolment. Presence of hypertension was defined by a combination of the use of two or more antihypertensive medications within 180 days prior to study enrolment or self-reported hypertension, which has a PPV of 80.0% and a specificity of 94.7% to predict hypertension [28, 29]. Statin use at study enrolment was obtained from a combination of self-reported use and filled prescriptions. Hypercholesterolaemia was defined by self-reported hypercholesterolaemia or self-reported statin-use. Prescriptions were identified by ATC codes in the Danish National Prescription Registry if they were claimed within 180 days prior to enrolment in the study (from 1994 onwards). All ATC codes used are presented in Supplemental Table 2.

### Statistical analysis

Baseline characteristics of the cohort are presented overall, and across quintiles of vegetable nitrate intake. First, a cross-sectional linear regression analysis was performed to investigate the association between vegetable and non-vegetable nitrate intake in quintiles and baseline SBP and DBP, both in the whole population and stratified by use of antihypertensive medication. Second, participants’ time-to-events were based on a maximum of 23 years of follow-up from the date of enrollment until the date of death, emigration, event of interest, or end of follow-up (August 2017), whichever came first. Nonlinear relationships were examined using restricted cubic splines, with hazard ratios (HRs) based on Cox proportional hazards models. All HRs and 95% confidence intervals (CIs) were obtained from the model with the exposure fitted as a continuous variable through a restricted cubic spline; HR estimates are reported for the median intake in each quintile with the first quintile median as the reference point and are graphed over a fine grid of x values. Cox proportional hazards assumptions were tested using log–log plots of the survival function versus time and assessed for parallel appearance, with no violation found. Our main models of adjustment were: Model 1a adjusting for age and sex; Model 1b adjusting for all covariates in Model 1a plus BMI, smoking status (current/former/never), physical activity (total daily metabolic equivalent), social economic status (income), marital status, education, pure alcohol intake (g/day), hypercholesterolemia (yes/no), and prevalent disease (diabetes, chronic obstructive pulmonary disease, chronic kidney disease, and cancer); entered into the model separately); Model 2 adjusting for all covariates in Model 1b plus energy; and Model 3: adjusting for all covariates in Model 2 plus intakes (g/day) of fish, red meat, polyunsaturated fatty acids, monounsaturated fatty acids, saturated fatty acids, and all fruit. Covariates were chosen a priori using expert and prior knowledge of potential confounders of nitrate intake and CVD. Additionally, standard logistic regression models were used to obtain the 20-year absolute risk estimates of incident CVD and CVD subtypes.

Analyses were further stratified by sex, BMI (above/below 30 kg/m^2^), alcohol intake (above/below 20 g/day), smoking status (ever/never), and by tertiles of total vegetable intake. When stratifying by alcohol intake and BMI, all participants with an alcohol intake of zero (n = 1180) and a BMI < 18.5 (n = 426) were excluded due to potential underlying pathologies or habits that may increase CVD risk. Stratification cut-off points of 20 g alcohol/day and a BMI of 30 kg/m^2^ were chosen as risk of disease is higher beyond these levels [30, 31]. We tested for interactions using chi-squared tests comparing nested standard cox proportional hazards models, using quintiles of vegetable nitrate intake.

The extent to which the association between vegetable nitrate intake and CVD was mediated by baseline SBP was quantified through natural direct and indirect effects [32], using a Cox proportional hazards model in the medflex package for R [33].

Analyses were undertaken using STATA/IC 14.2 (StataCorp LLC) and R statistics (R Core Team, 2019)[34].

## Results

The 53,150 Danish participants included in the present study had a median [IQR] age of 56 [52–60] years at entry and a median [IQR] follow-up of 21 [15–22] years. Total median [IQR] vegetable nitrate intake was 59 mg/day [35–98] and non-vegetable nitrate intake was 15 mg/day [12–18]. Major individual vegetables that contributed to vegetable nitrate intake were lettuce (41%), potato (22%), celery (10%), carrot (5%), and spinach (3%). During follow-up, 14,088 participants were hospitalized for any CVD, 5327 for IHD, 2,885 for ischemic stroke, 709 for haemorrhagic stroke, 3081 for heart failure, 1867 for PAD, and 6748 for AF, with 4699 (8.84%) participants receiving a diagnosis of two or more types of CVD.

### Baseline characteristics

Compared to participants in the lowest quintile of vegetable nitrate intake, those in the highest quintile were more likely to be female, have a slightly lower BMI, be more physically active, have never smoked, have a higher degree of education, have a higher income, and were less likely to hypertensive and more likely to be diabetic. Furthermore, those with a high vegetable nitrate intake tended to consume more fish, vegetables, fruit, fibre, flavonoids, and less processed meat than those with a low vegetable nitrate intake (Table 1).

**Table 1:** Baseline characteristics of study population

	Total population n = 53 150	Vegetable nitrate intake quintiles				
		Q1 n = 10 630	Q2 n = 10 630	Q3 n = 10 630	Q4 n = 10 630	Q5 n = 10 630
Vegetable nitrate intake (mg/day)	59 [35, 98]	23 [18, 28]	39 [35, 43]	59 [52, 68]	92 [86, 98]	141 [118, 168]
Non-vegetable nitrate intake (mg/day)	15 [12, 18]	12 [10, 15]	14 [12, 18]	15 [12, 19]	15 [12, 18]	16 [13, 20]
Sex (male)	46.4 (24 682)	47.2 (5 014)	51.5 (5 475)	47.5 (5 044)	42.8 (4 546)	43.3 (4 603)
Age (years)	56 [52, 60]	56 [53, 60]	56 [52, 60]	55 [52, 60]	55 [52, 59]	55 [52, 59]
BMI (kg/m^2^)	26 [23, 28]	26 [24, 29]	26 [24, 28]	26 [23, 28]	25 [23, 28]	25 [23, 28]
MET score	56 [37, 84]	50 [32, 78]	55 [36, 84]	58 [39, 87]	57 [38, 84]	61 [41, 90]
*Smoking status*						
Never	36.0 (19 122)	29.9 (3 182)	33.4 (3 555)	36.9 (3 921)	40.0 (4 254)	39.6 (4 210)
Former	28.3 (15 064)	23.1 (2 460)	27.3 (2 903)	28.7 (3 055)	30.2 (3 208)	32.3 (3 438)
Current	35.7 (18 964)	46.9 (4 988)	39.2 (4 172)	34.4 (3 654)	29.8 (3 168)	28.1 (2 982)
*Education*						
≤ 7 years	32.3 (17 164)	45.1 (4 790)	37.0 (3 931)	31.8 (3 383)	25.4 (2 697)	22.2 (2 363)
8 – 10 years	46.4 (24 653)	43.5 (4 626)	47.5 (5 045)	48.1 (5 110)	49.0 (5 207)	43.9 (4 665)
≥ 11 years	21.3 (11 310)	11.4 (1 210)	15.5 (1 648)	20.0 (2 130)	25.6 (2 725)	33.8 (3 597)
*Marital status*						
Married	71.0 (37 737)	62.4 (6 631)	71.4 (7 591)	71.6 (7 611)	76.0 (8 075)	73.7 (7 829)
Unmarried	28.2 (14 991)	36.7 (3 901)	27.7 (2 946)	27.6 (2 937)	23.4 (2 489)	25.6 (2 718)
Unknown	0.8 (422)	0.9 (98)	0.9 (93)	0.8 (82)	0.6 (66)	0.8 (83)
*Mean household income*						
≤ 394 700 DKK/year	24.3 (12 938)	34.3 (3 650)	25.3 (2 688)	24.3 (2 578)	18.4 (1 954)	19.5 (2 068)
394 701 – 570 930 DKK/year	24.8 (13 194)	28.7 (3 056)	27.5 (2 924)	24.4 (2 599)	22.3 (2 366)	21.2 (2 249)
570 931 – 758 297 DKK/year	25.2 (13 399)	22.4 (2 384)	26.6 (2 824)	26.6 (2 831)	26.9 (2 858)	23.5 (2 502)
> 758 297 DKK/year	25.6 (13 619)	14.5 (1 540)	20.6 (2 194)	24.7 (2 622)	32.5 (3 452)	35.9 (3 811)
Systolic blood pressure (mm Hg)	138 [124, 152]	140 [127, 155]	139 [126, 153]	137 [124, 152]	136 [124, 150]	136 [123, 150]
Diastolic blood pressure (mm Hg)	83 [76, 90]	84 [76, 91]	83 [76, 90]	83 [76, 89]	82 [76, 89]	81 [75, 88]
Hypertensive	15.5 (8 212)	16.5 (1 753)	15.4 (1 635)	15.7 (1 666)	15.2 (1 617)	14.5 (1 541)
Hypercholesterolemic	6.3 (3 335)	6.0 (636)	6.6 (703)	6.4 (676)	6.2 (656)	6.2 (664)
*Comorbidities*						
Diabetes	1.8 (979)	1.7 (183)	1.5 (155)	1.9 (198)	1.5 (156)	2.7 (287)
CKD	0.3 (174)	0.3 (33)	0.3 (32)	0.4 (40)	0.4 (38)	0.3 (31)
COPD	1.4 (733)	1.9 (199)	1.6 (165)	1.3 (135)	1.1 (118)	1.1 (116)
Cancer	0.4 (222)	0.5 (54)	0.5 (49)	0.5 (48)	0.3 (34)	0.3 (37)
*Medication use*						
Diabetes medication	1.1 (569)	0.9 (95)	0.8 (90)	1.1 (116)	0.9 (94)	1.6 (174)
Antihypertensive	11.6 (6 155)	12.5 (1 327)	11.6 (1 238)	11.6 (1 232)	11.4 (1 207)	10.8 (1 151)
Statin	1.2 (612)	1.1 (113)	1.2 (124)	1.2 (127)	1.1 (117)	1.2 (131)
*HRT (% of women)*						
Never	54.4 (15 484)	53.7 (3 015)	53.7 (2 770)	54.8 (3 061)	55.0 (3 347)	54.6 (3 291)
Current	30.1 (8 569)	29.4 (1 652)	30.7 (1 581)	29.5 (1 648)	30.3 (1 841)	30.6 (1 847)
Former	15.4 (4 384)	16.8 (942)	15.4 (793)	15.6 (873)	14.6 (890)	14.7 (886)
NSAID	32.1 (16 938)	31.5 (3 321)	31.4 (3 310)	32.4 (3 433)	32.5 (3 440)	32.5 (3 434)
Aspirin	12.1 (6 423)	13.0 (1 382)	(1 295) 12.2	12.1 (1 283)	11.6 (1 232)	11.6 (1 231)
*Dietary characteristics*						
Energy (kj/d)	9 493 [7 849, 11 360]	8 362 [6 836, 10 041]	9 369 [7 822, 11 127]	9 840 [8 178, 11 805]	9 593 [8 048, 11 350]	10 343 [8 655, 12 199]
Total fish intake (g/day)	38 [25, 55]	29 [19, 43]	37 [25, 52]	41 [28, 58]	40 [27, 56]	44 [30, 63]
Red meat intake (g/day)	78 [56, 107]	70 [51, 94]	81 [60, 109]	82 [59, 114]	78 [57, 105]	79 [56, 111]
Processed meat intake (g/day)	24 [14, 40]	26 [15, 42]	27 [17, 43]	25 [14, 41]	23 [14, 36]	21 [12, 36]
Dietary fibre intake (g/day)	20 [16, 25]	15 [12, 19]	19 [15, 23]	22 [18, 26]	21 [17, 26]	25 [21, 30]
Saturated FA (g/day)	31 [24, 39]	29 [22, 37]	31 [24, 39]	32 [25, 41]	31 [24, 39]	32 [25, 41]
Polyunsaturated FA (g/day)	13 [10, 17]	11 [8, 14]	12 [10, 16]	14 [11, 18]	14 [11, 18]	16 [12, 20]
Monounsaturated FA (g/day)	27 [21, 35]	25 [19, 32]	28 [22, 35]	28 [22, 36]	27 [21, 34]	28 [22, 36]
Fruit intake (g/day)	172 [95, 282]	116 [52, 203]	152 [82, 251]	184 [113, 294]	188 [116, 293]	227 [138, 356]
Vegetable intake (g/day)	162 [105, 232]	72 [51, 99]	119 [93, 151]	173 [139, 217]	192 [158, 235]	284 [233, 350]
Total flavonoid intake (mg/day)	498 [289, 808]	342 [192, 618]	427 [257, 716]	516 [311, 814]	561 [334, 857]	649 [393, 986]
Alcohol intake (g/day)	13 [6, 31]	11 [3, 31]	13 [6, 31]	13 [6, 29]	14 [7, 31]	14 [7, 32]

Data expressed as median [IQR] or % (n)

BMI, body mass index; CKD, chronic kidney disease; COPD, common obstructive pulmonary disease; DKK, Danish Krone; FA, fatty acids; HRT, hormone replacement therapy, MET, metabolic equivalent; NSAID, Nonsteroidal anti-inflammatory drug

### Cross sectional analysis of vegetable and non-vegetable nitrate intake and blood pressure

At baseline, vegetable nitrate intake was inversely associated with SBP and DBP in a linear dose–response manner. After multivariable adjustments and compared to participants in quintile 1, participants in quintile 5 had a 2.58 mmHg lower SBP (95% CI − 3.12, − 2.05) and 1.38 mmHg lower DBP (− 1.66, − 1.10) (Table 2). The inverse association between vegetable nitrate intake and BP was present in individuals both on and not on antihypertensive medication at baseline (Supplemental Table 3 and 4). No association was observed between non-vegetable nitrate intake and BP (Supplemental Table 5).

**Table 2:** Association between vegetable nitrate intake (mg/day) and blood pressure

	Vegetable nitrate intake quintiles					
	Q1 n = 10,630	Q2 n = 10,630	Q3 n = 10,630	Q4 n = 10,630	Q5 n = 10,630	P for trend
Systolic blood pressure	Ref	− 1.08 (− 1.59, − 0.56)	− 1.85 (− 2.37, − 1.33)	− 2.06 (− 2.58, − 1.53)	− 2.58 (− 3.12, − 2.05)	< 0.001
Diastolic blood pressure	Ref	− 0.65 (− 0.92, − 0.39)	− 0.92 (− 1.19, − 0.39)	− 1.08 (− 1.36, − 0.80)	− 1.38 (− 1.66, − 1.10)	< 0.001

Results are analysed by linear regression and are adjusted for age, sex, BMI, smoking status (current/former/never), physical activity (total daily metabolic equivalent), pure alcohol intake (g/day), social economic status (income), marital status, hypercholesterolemia (yes/no), education, and prevalent disease (diabetes, chronic obstructive pulmonary disease, chronic kidney disease, and cancer; entered into the model separately). Results are presented as linear coefficient (95% CI)

### Association between vegetable nitrate intake and incident CVD and CVD subtypes

**Fig. 1:**
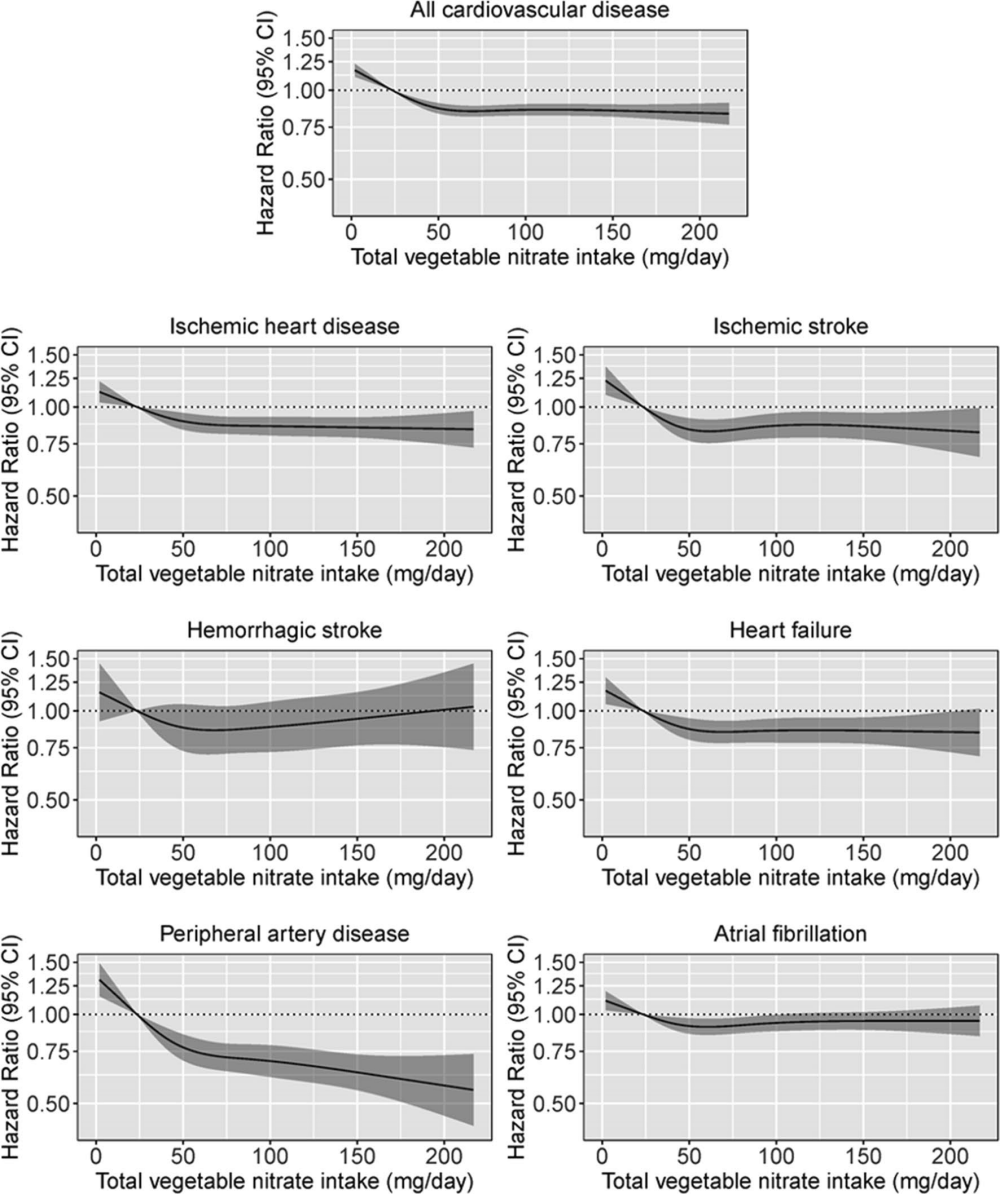
Cubic spline curves describing the association between vegetable nitrate intake and CVD incidence and the incidence of CVD subtypes; ischemic heart disease, ischemic stroke, hemorrhagic stroke, heart failure, peripheral artery disease and atrial fibrillation. Hazard ratios are based on Cox proportional hazards models adjusted for age, sex, BMI, smoking status, physical activity, alcohol intake, education, social economic status (income), marital status, hypercholesterolemia, diabetes, COPD, chronic kidney disease and cancer and are comparing the specific level of vegetable nitrate intake (horizontal axis) to the median intake for participants in the lowest intake quintile (23 mg/day)

Vegetable nitrate intake was non-linearly associated with CVD hospitalizations (Model 1b; Fig. 1). On a relative scale, participants with a moderate vegetable nitrate intake (quintile 3) had a 15% lower risk of CVD [HR (95% CI): 0.85 (0.82, 0.89)] after multivariable adjustments (Model 1b; Table 3) and compared to participants in quintile 1. Comparable risk estimates were observed for participants in higher intake quintiles. On an absolute scale, the difference (quintile 3–quintile 1) in the 20-year estimated risk of being hospitalised for CVD was 2.79% for males and 1.88% for females (Supplemental Table 6 and 7).

**Table 3:** Hazard ratios of incident CVD and CVD subtypes by quintiles of vegetable nitrate intake

	Vegetable nitrate intake quintiles				
	Q1 (n = 10,630)	Q2 (n = 10,630)	Q3 (n = 10,630)	Q4 (n = 10,630)	Q5 (n = 10,630)
Intake (mg/day)*	23 [18–28]	39 [35–43]	59 [52–68]	92 [86–98]	141 [118–168]
*CVD*					
No. events	3309	3037	2725	2456	2561
*HR (95% CI)*					
Model 1a	Ref	0.86 (0.84, 0.89)	0.77 (0.74, 0.80)	0.74 (0.71, 0.77)	0.73 (0.69, 0.76)
Model 1b	Ref	0.91 (0.88, 0.94)	0.85 (0.82, 0.89)	0.86 (0.82, 0.89)	0.86 (0.82, 0.90)
Model 2	Ref	0.91 (0.88, 0.93)	0.85 (0.82, 0.89)	0.85 (0.82, 0.89)	0.85 (0.81, 0.90)
Model 3	Ref	0.90 (0.87, 0.93)	0.84 (0.81, 0.88)	0.85 (0.82, 0.89)	0.86 (0.82, 0.91)
*IHD*					
No. events	1,279	1,150	1,045	846	917
*HR (95% CI)*					
Model 1a	Ref	0.87 (0.83, 0.91)	0.77 (0.72, 0.82)	0.70 (0.66, 0.75)	0.68 (0.63, 0.73)
Model 1b	Ref	0.93 (0.88, 0.97)	0.88 (0.82, 0.94)	0.86 (0.80, 0.93)	0.85 (0.79, 0.92)
Model 2	Ref	0.93 (0.89, 0.98)	0.88 (0.82, 0.95)	0.87 (0.80, 0.93)	0.86 (0.79, 0.93)
Model 3	Ref	0.92 (0.88, 0.97)	0.87 (0.81, 0.94)	0.87 (0.80, 0.93)	0.87 (0.79, 0.94)
*Ischemic stroke*					
No. events	714	613	518	509	531
*HR (95% CI)*					
Model 1a	Ref	0.84 (0.78, 0.89)	0.75 (0.68, 0.82)	0.74 (0.68, 0.81)	0.74 (0.67, 0.81)
Model 1b	Ref	0.88 (0.83, 0.94)	0.83 (0.76, 0.91)	0.86 (0.78, 0.94)	0.87 (0.78, 0.96)
Model 2	Ref	0.89 (0.83, 0.95)	0.83 (0.76, 0.92)	0.86 (0.78, 0.95)	0.87 (0.79, 0.97)
Model 3	Ref	0.87 (0.81, 0.93)	0.81 (0.74, 0.89)	0.85 (0.77, 0.94)	0.86 (0.77, 0.97)
*Haemorrhagic stroke*					
No. events	170	127	151	121	140
*HR (95% CI)*					
Model 1a	Ref	0.88 (0.77, 1.01)	0.81 (0.67, 0.98)	0.81 (0.68, 0.97)	0.87 (0.71, 1.05)
Model 1b	Ref	0.91 (0.80, 1.04)	0.86 (0.71, 1.04)	0.87 (0.72, 1.06)	0.93 (0.76, 1.14)
Model 2	Ref	0.90 (0.79, 1.03)	0.84 (0.69, 1.02)	0.85 (0.70, 1.03)	0.90 (0.73, 1.11)
Model 3	Ref	0.91 (0.80, 1.05)	0.86 (0.71, 1.05)	0.87 (0.71, 1.07)	0.93 (0.74, 1.16)
*Heart failure*					
No. events	778	704	575	497	527
*HR (95% CI)*					
Model 1a	Ref	0.83 (0.78, 0.88)	0.71 (0.66, 0.78)	0.67 (0.61, 0.73)	0.65 (0.59, 0.72)
Model 1b	Ref	0.91 (0.85, 0.96)	0.85 (0.78, 0.93)	0.86 (0.78, 0.94)	0.86 (0.78, 0.95)
Model 2	Ref	0.90 (0.85, 0.96)	0.84 (0.77, 0.92)	0.84 (0.77, 0.93)	0.84 (0.76, 0.94)
Model 3	Ref	0.88 (0.83, 0.94)	0.82 (0.74, 0.89)	0.83 (0.75, 0.91)	0.83 (0.75, 0.93)
*PAD*					
No. events	539	417	363	268	255
*HR (95% CI)*					
Model 1a	Ref	0.76 (0.70, 0.82)	0.60 (0.54, 0.67)	0.52 (0.47, 0.58)	0.47 (0.41, 0.53)
Model 1b	Ref	0.84 (0.78, 0.91)	0.74 (0.67, 0.83)	0.70 (0.62, 0.79)	0.65 (0.57, 0.74)
Model 2	Ref	0.85 (0.78, 0.91)	0.75 (0.67, 0.83)	0.71 (0.62, 0.80)	0.65 (0.57, 0.75)
Model 3	Ref	0.84 (0.77, 0.91)	0.74 (0.66, 0.83)	0.73 (0.64, 0.82)	0.69 (0.59, 0.80)
*AF*					
No. events	1457	1422	1315	1237	1317
*HR (95% CI)*					
Model 1a	Ref	0.91 (0.87, 0.95)	0.85 (0.80, 0.91)	0.85 (0.80, 0.91)	0.87 (0.81, 0.92)
Model 1b	Ref	0.94 (0.90, 0.98)	0.91 (0.85, 0.97)	0.93 (0.87, 0.99)	0.95 (0.89, 1.01)
Model 2	Ref	0.93 (0.89, 0.98)	0.90 (0.84, 0.96)	0.92 (0.86, 0.98)	0.94 (0.88, 1.01)
Model 3	Ref	0.93 (0.89, 0.97)	0.89 (0.83, 0.95)	0.91 (0.85, 0.97)	0.92 (0.86, 1.00)

Hazard ratios (95% CI) for incident CVD and CVD subtypes during 23 years of follow-up obtained from restricted cubic splines based on Cox proportional hazards models. Model 1a adjusted for age and sex; Model 1b adjusted for all covariates in Model 1a plus BMI, smoking status (current/former/never), physical activity (total daily metabolic equivalent), pure alcohol intake (g/day), social economic status (income), marital status, hypercholesterolemia (yes/no), education, and prevalent disease (diabetes, chronic obstructive pulmonary disease, chronic kidney disease, and cancer; entered into the model separately); Model 2 adjusted for all covariates in Model 1b plus energy; Model 3: adjusted for all covariates in Model 2 plus intakes (g/day) of fish, red meat, polyunsaturated fatty acids, monounsaturated fatty acids, saturated fatty acids, and all fruit

^*^Median; range in parentheses (all such values)

For CVD subtypes, after multivariable adjustments (Model 1b; Table 3) and compared to participants in quintile 1, participants in quintile 3 had a 12% lower risk of IHD [0.88 (0.82, 0.94)], 17% lower risk of ischemic stroke [0.83 (0.76, 0.91)], 15% lower risk of heart failure [0.85 (0.78, 0.93)], and a 26% lower risk of PAD [0.74 (0.67, 0.83)]. The HR remained constant for higher levels of exposure except for PAD for which the lowest risks were observed for those in quintile 5 [Q5; 0.65 (0.57, 0.74)]. There was no clear association between vegetable nitrate intake and haemorrhagic stroke or AF. The association between vegetable nitrate intake and CVD hospitalizations was still observed after adjusting for potential dietary confounders (Model 3; Table 3). The absolute risk differences for the CVD subtypes are presented in Supplemental Table 7 and 8.

### Mediation analysis

Mediation analyses showed that baseline SBP explained 21.9% of the total association between vegetable nitrate intake and incident CVD.

### Associations between non-vegetable nitrate intake and incident CVD and CVD subtypes

The association between non-vegetable nitrate intake and incident CVD was non-linear (Supplemental Figure 2). After multivariable adjustments (Model 1b) and compared to participants in quintile 1, participants in quintile 3 had a 6% lower risk of a CVD hospitalization [0.94 (0.90, 0.98)]. Of the secondary outcomes, participants in quintile 3 had a 19% lower risk of haemorrhagic stroke [0.81 (0.70, 0.94)], and a 15% lower risk for PAD [0.85 (0.78, 0.94)] (Supplemental Table 8). As observed with vegetable nitrate intake, the HR remained constant for higher levels of exposure except for PAD for which the lowest risks were observed for those in quintile 5 [Q5; 0.79 (0.70, 0.90)].

### Validated case analysis

Using only medically reviewed and validated cases, 3,430 participants were admitted to hospital for an MI, ischemic stroke, or PAD. Vegetable nitrate intake was inversely associated with validated cases of MI, ischemic stroke and PAD (Supplemental Figure 3).

### Stratified analyses

**Fig. 2:**
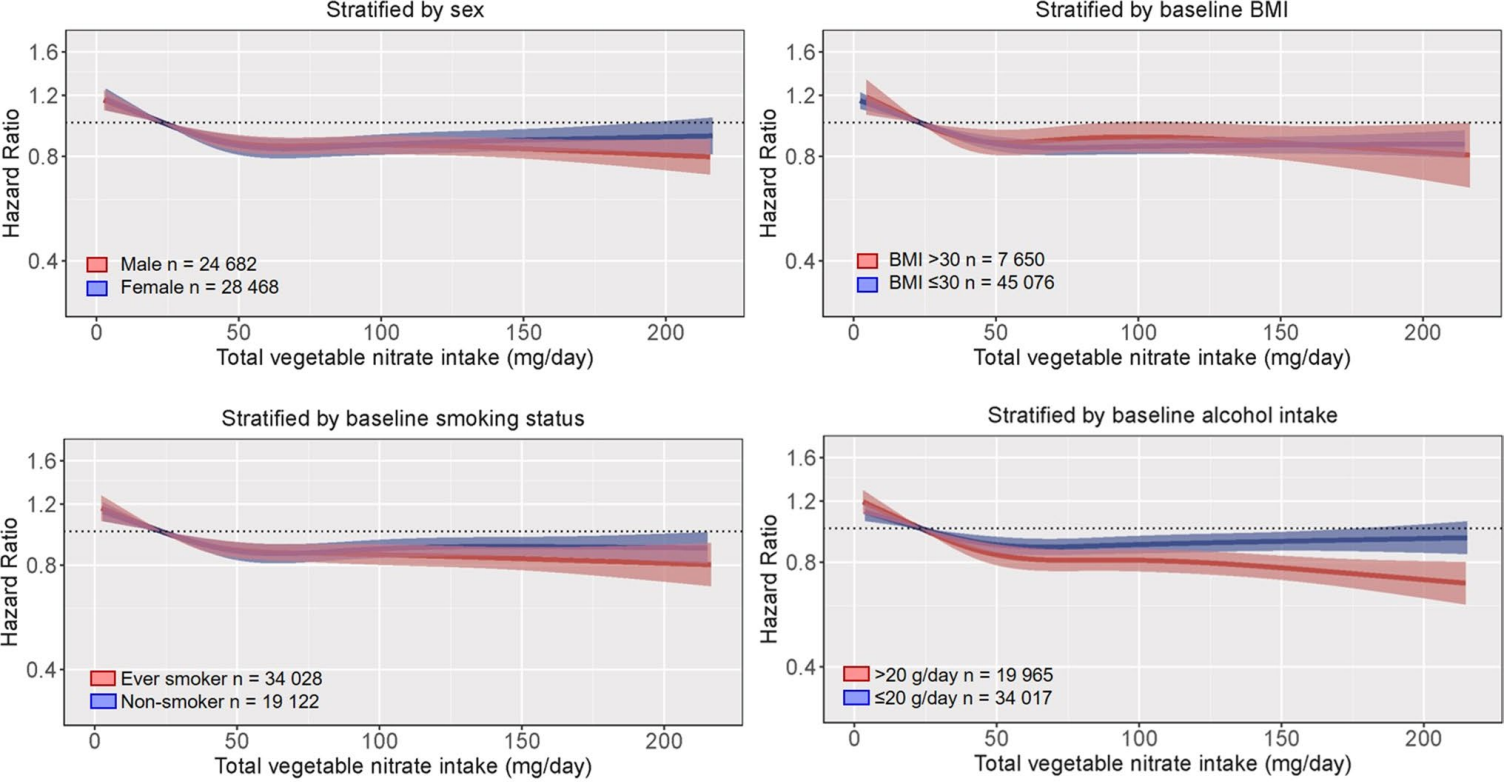
Multivariable-adjusted association between total vegetable nitrate intake and incident CVD stratified by sex, baseline BMI, smoking status, or alcohol intake. Hazard ratios are based on Cox proportional hazards models and are comparing the specific level of vegetable nitrate intake (horizontal axis) to the median intake for participants in the lowest intake quintile (23 mg/day). All analyses were adjusted for age, sex, BMI, smoking status, physical activity (total daily metabolic equivalent), pure alcohol intake (g/day), social economic status (income), marital status, hypercholesterolemia (yes/no), education, and prevalent disease (diabetes, chronic obstructive pulmonary disease, chronic kidney disease, and cancer; entered into the model separately)

The association between vegetable nitrate and incident CVD was modified by alcohol intake (p for interaction < 0.001; Fig. 2); the association was stronger in high alcohol consumers [Q5; 0.78 (0.72, 0.84)] than in low alcohol consumers [Q5; 0.92 (0.86, 0.97)]. The association between vegetable nitrate intake and incident CVD did not appear to be modified by sex, baseline BMI, and smoking status (*p* for interaction > 0.05 for all).

To understand whether vegetable nitrate intake is only a marker of higher vegetable intake, we stratified our analysis by tertiles of total vegetable intake. There still appeared to be an association between vegetable nitrate intakes and a lower risk of CVD in all tertiles of total vegetable intake (Supplemental Table 9).

## Discussion

In this prospective cohort study of 53,150 Danish people without CVD at baseline, we found that a higher vegetable nitrate intake was associated with a lower SBP and DBP at baseline. Furthermore, we observed that a moderate intake of vegetable nitrate was inversely associated with incident CVD, with no additional benefits observed for higher intakes. This association was partially mediated by SBP and was stronger in individuals with high alcohol consumption than in those with a low to moderate alcohol consumption. For CVD subtypes, individuals with moderate to high vegetable nitrate intakes had a lower risk of atherosclerotic CVD hospitalisations, namely IHD, ischemic stroke, heart failure, and PAD, but not for other CVD hospitalisations, namely haemorrhagic stroke or AF.

We observed that participants in the highest quintile of vegetable nitrate intake had a 2.58 mmHg lower SBP and a 1.38 mmHg lower DBP at baseline, compared to those in the lowest quintile. In the Atherosclerosis Risk in Communities Study, a 2 mmHg lower SBP was associated with 17.9 fewer CHD events, 9.6 fewer stroke events, and 26.6 fewer heart failure events per 100,000 person-years [35]. Framingham Heart Study investigators observed that a 2 mmHg lower DBP was associated with a 6% lower risk of CHD and a 15% lower risk of stroke in men and women aged 35–64 years [36]. Our results support findings from short-term clinical trials, and meta-analyses of these trials, demonstrating a benefit of nitrate intake on BP [37]. However, not all clinical trials have observed a reduction in BP with nitrate intake. The majority of clinical trials observe reductions in BP with nitrate intake in normotensive individuals, however effects in individuals with high-normal BP and hypertension are less clear [37]. The largest clinical trial to date [38], did not observe a reduction in BP in 243 older subjects with elevated BP after 5 weeks nitrate intake. Interestingly, we observed an inverse association between vegetable nitrate intake and BP in individuals both on and not on anti-hypertensive medication.

To our knowledge, the association between vegetable nitrate intake and long-term cardiovascular outcomes has only been investigated in four cohorts. In the Perth Longitudinal Study of Aging in Women, vegetable nitrate intake was associated with a 21% lower risk of atherosclerotic vascular disease mortality (238 incident cases) and 17% lower risk of an ischemic cerebrovascular disease event (186 incident cases) per 1 SD (~ 30 mg/day) higher intake [9, 10]. In participants of the Australian Blue Mountains Eye Study, those in the highest quartile of vegetable nitrate intake had a 27% lower risk of CVD mortality (188 incident cases) compared to those in the lowest intake quartile [12]. In women of Australian Longitudinal Study on Women’s Health, those in the highest quartile of vegetable nitrate intake had a 27% lower risk of developing self-reported CVD-related complications (1951 incident cases), compared to those in the lowest intake quartile [11]. In the American Nurses’ Health Study, women in the highest quintile of vegetable nitrate intake had weak evidence for a lower risk of either fatal or non-fatal CHD (2267 incident cases), compared to those in the lowest quintile [13]. In the present study, we observed that a higher vegetable nitrate intake was associated with 15% lower risk of incident CVD compared to participants in the lowest quintile. Interestingly, no further reduction in risk of incident CVD was observed for intakes above ~ 60 mg/day (~ 1 cup of green leafy vegetables per day). This is in agreement with results from the previously described prospective studies. In the Perth Longitudinal Study of Aging in Women, the inverse relationship with atherosclerotic vascular disease mortality and ischemic cerebrovascular events plateaued at vegetable nitrate intakes of 53–76 mg/day [9, 10]. Similarly, in the Blue Mountains Eye Study no added benefit on risk of CVD mortality was observed above intakes of 70 – 100 mg/day [12]. In the Australian Longitudinal Study on Women’s Health a threshold effect was not observed, however those in the highest quintile of vegetable nitrate intake had a mean intake of 79 mg/day [11]. Clinical trials investigating the vascular effects of nitrate intake report acute effects at doses ranging from 68 to 1488 mg and chronic effects at doses ranging from 155 to 1200 mg [37].

A lower risk of IHD, ischemic stroke, heart failure, and PAD, but not haemorrhagic stroke or AF, was observed in individuals with moderate to high vegetable nitrate intakes. The primary mechanism through which dietary nitrate may impact cardiovascular health is via augmentation of NO [2]. Endothelium-derived NO maintains vascular tone influencing blood flow and BP [2]. In the present study, baseline SBP explained 21.9% of the total association between vegetable nitrate intake and incident CVD, indicating that the effects of nitrate on BP reported in clinical trials, translate to a lower risk of incident CVD, but also that other mechanisms are in play. Hypertension is associated with ischemic stroke, CHD, heart failure, AF, and PAD [39]. It may be that the decrease in SBP or DBP may not be enough to mitigate the risk of AF and haemorrhagic stroke, however, the impact of BP reduction on the risk of AF is still unclear [40].

We demonstrated that the association between vegetable nitrate intake and incident CVD was stronger in those who consume, on average, more than 20 g (2 standard drinks) of alcohol per day compared to those who consume less than 20 g of alcohol per day. Heavy alcohol consumption increases the risk of CVD [41] and detrimental effects on a number of physiological parameters, including blood pressure [31] and endothelial function [42] have been reported. Our findings suggest the vegetable nitrate intake may partially mitigate these harmful effects.

This study has several strengths. The large number of incident cases and 23-year duration of follow-up, with limited loss to follow-up, afforded us the statistical power to examine associations in subpopulations at higher risk of CVD. Our findings were supported by a validated case analysis. A comprehensive database detailing the nitrate content of vegetables was used to calculate vegetable nitrate intake [3] in combination with a validated FFQ that included average intakes of foods commonly consumed in Denmark over the previous 12 months. The same up to date database was used in three previous smaller prospective studies [9–12], with results consistent with the current analysis. However, an older less comprehensive nitrate database was used in the Nurses Health Study [13]. This may partially explain the apparent weaker associations observed in this study.

Several limitations should also be considered. As this is an observational study, we are unable to infer causality or rule out residual or unmeasured confounding factors. Although the association between higher nitrate intake and lower risk of a hospital admission for CVD was still present after adjustment for lifestyle factors and other indicators of a healthy diet, as well as amongst participants in the highest vegetable intake tertile, determining the effect of nitrate alone can only be achieved through intervention studies. Furthermore, we were unable to investigate potential interactions with known inhibitors of the nitrate-nitrite-NO pathway, including antibacterial mouthwash and proton pump inhibitors. The measurement of BP was not gold standard, but any measurement error would have reduced our power to detect an association. Finally, participants in this study were most likely Caucasian meaning that caution should be taken when extrapolating these findings to other races and ethnicities.

## Conclusion

In this Danish prospective cohort study, we observed an inverse association between vegetable nitrate intakes, up to 60 mg/day, and hospital admissions for CVD. More specifically, moderate to high nitrate intakes were associated with a lower risk of IHD, ischemic stroke, PAD, and HF. A higher vegetable nitrate intake was also associated with a lower baseline SBP and DBP. Our results suggest that ensuring the consumption of nitrate-rich vegetables, corresponding to ~ 1 cup of green leafy vegetables, may lower the risk of CVD.

## Supplementary Information

Below is the link to the electronic supplementary material.

Supplementary file 1 (DOCX 3220 KB)

## Data Availability

Data described in the manuscript, code book, and analytic code will be made available upon request pending application and approval by the Diet, Cancer and Health Steering Committee at the Danish Cancer Society.
