# Supplementary material for: Vegetable nitrate intake, blood pressure and incident cardiovascular disease: Danish Diet, Cancer, and Health Study
Source: Eur J Epidemiol. 2021 Apr 21;36(8):813–25. doi: 10.1007/s10654-021-00747-3 (PMC8416839; doi:10.1007/s10654-021-00747-3)
Supplement: Supplementary file 1 — (DOCX 3220 KB) [file 10654_2021_747_MOESM1_ESM.docx]

**Vegetable nitrate intake, blood pressure and incident cardiovascular disease: Danish Diet, Cancer, and Health Study.**

Catherine P Bondonno PhD^1,2^*****, Frederik Dalgaard MD^3^*, Lauren C Blekkenhorst PhD^1,2^, Kevin Murray PhD^4^, Joshua R Lewis PhD^1,2,5^, Kevin D Croft PhD^6^, Cecilie Kyrø PhD^7^, Christian Torp-Pedersen, MD, DMS^8^, Gunnar Gislason PhD^3,9,10^, Anne Tjønneland MD, PhD, DMSc^7,11^, Kim Overvad PhD^12,13^, Nicola P Bondonno PhD^1,4†^, Jonathan M Hodgson PhD^1,2†^.

^1^ Institute for Nutrition Research, School of Medical and Health Sciences, Edith Cowan University, Perth, Australia; (CPB: 0000-0001-8509-439X; LCB: 0000-0003-1561-9052; JRL: 0000-0003-1003-8443; NPB: 0000-0001-5905-444X; JMH: 0000-0001-6184-7764)

^2^ Medical School, The University of Western Australia, Royal Perth Hospital, Perth, Western Australia, Australia;

^3^ Department of Cardiology, Herlev & Gentofte University Hospital, Copenhagen, Denmark; (FD: 0000-0002-7287-4191; GG: 0000-0002-0548-402X)

^4^ School of Population and Global Health, University of Western Australia, Australia; (KM: 0000-0002-8856-6046)

^5^Centre for Kidney Research, Sydney Medical School, School of Public Health, The University of Sydney, Sydney, Australia

^6^ School of Biomedical Sciences, University of Western Australia, Royal Perth Hospital, Perth, Western Australia, Australia; (KDC: 0000-0003-1596-4913)

^7^ The Danish Cancer Society Research Center, Copenhagen, Denmark; (CK: 0000-0002-9083-8960; AT: 0000-0003-4385-2097)

^8^ Department of Clinical Investigation and Cardiology, Nordsjælland Hospital, Hillerød, Denmark; (CTP: 0000-0003-2892-6131)

^9^ The National Institute of Public Health, University of Southern Denmark, Odense, Denmark;

^10^ The Danish Heart Foundation, Copenhagen, Denmark;

^11^ Department of Public Health, Faculty of Health and Medical Sciences, University of Copenhagen, Copenhagen, Denmark;

^12^ Department of Public Health, Aarhus University, Aarhus, Denmark; (KO: 0000-0001-6429-7921)

^13^ Department of Cardiology, Aalborg University Hospital, Aalborg, Denmark.

* These authors contributed equally to this work

^†^ Co-senior author

**Address for correspondance** : Catherine P. Bondonno

School of Medical and Health Sciences, Edith Cowan University, Level 3, Royal Perth Hospital Research Foundation

Rear 50 Murray St, Perth Western Australia, Australia WA 6000

Tel: +61 8 9224 0339

Email: [c.bondonno@ecu.edu.au](mailto:c.bondonno@ecu.edu.au)

**Supplementary Material**


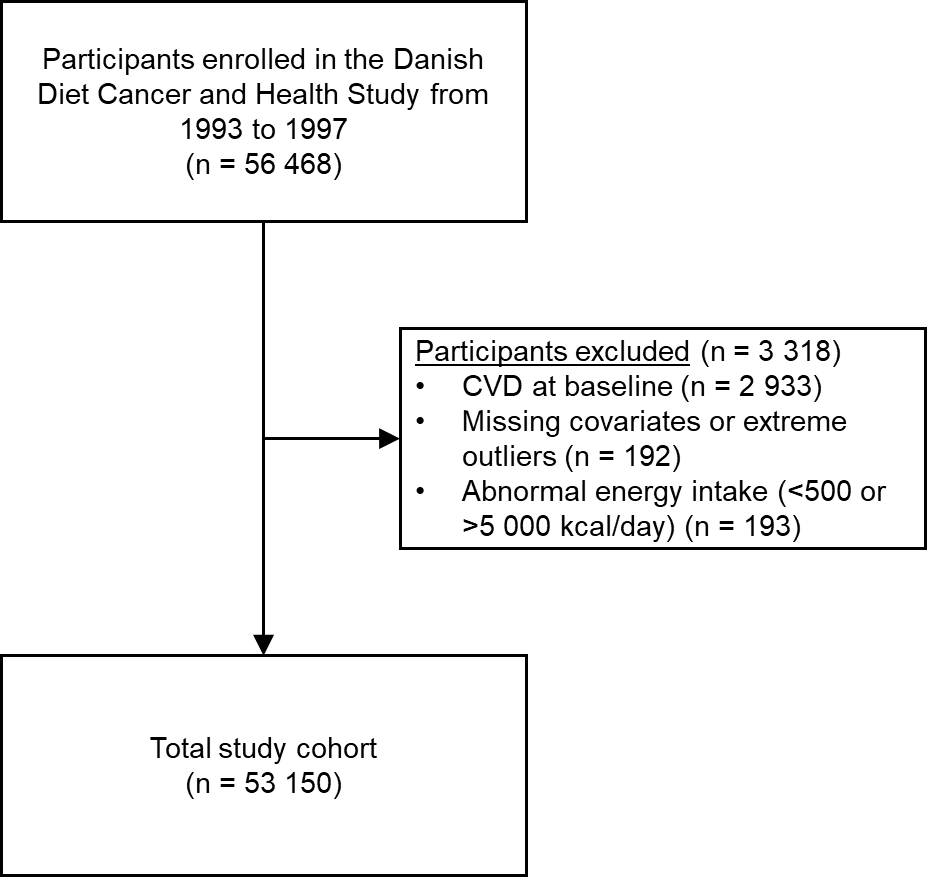
**Supplemental Figure 1**

**Supplemental Figure 1.** **Consort flow diagram**. CVD, cardiovascular disease.

| **Supplemental Table 1. International Classification of Disease codes used to determine validated cases of atherosclerotic cardiovascular disease** | | |
| --- | --- | --- |
| **Validated Diagnosis** | **ICD codes (ICD-8; ICD-10)** | **Follow-up** |
| Myocardial infarction | I21 | 10-02-1994 to 04-07-2013 |
| Peripheral artery disease | I702, I702A, I739A, I739B, I739C | 14-11-1994 to 07-12-2009 |
| Ischemic stroke | I63 | 04-03-1994 to 30-11-2009 |
| The methods for validating these cases have been published previously (1-3) . | | |

| **Supplemental Table 2.**  **Anatomical Therapeutic Chemical codes for antihypertensive medications** | |
| --- | --- |
| Use of antihypertensive medication was defined by the usage of any combination of at least two of the seven different drugs classes at the same time. | Non-Loop: Thiazides C02L, C02DA, C07B, C07D, C09XA52, C03A, C03EA;  Low-ceiling diuretics (excl. thiazides): C03B, C03X, C07C, C08G, C09BA, C09DA; potassium-sparing agents (spiron): C03D, C03E, C03EB  Loop: high-ceiling diuretics (Loop) C03C, C03EB  Antiadrenergic agents: C02A, C02B, C02C  Beta-blockers: C07A, C07B, C07C, C07D, C07F  Vasodilators: C02DB, C02DD, C02DG  Calcium channel blockers: C08, C09BB, C09DB  Renin angiotensin system inhibitors and angiotensin II receptor blockers: C09AA, C09BA, C09BB, C09CA, C09DA, C09DB, C09XA02, C09XA52 |
| Diabetes medication | A10A, A10B |
| Statins | C10AA |

| **Supplemental Table 3. Association between vegetable nitrate intake (mg/d) and blood pressure in people on antihypertensive medication** | | | | | | |
| --- | --- | --- | --- | --- | --- | --- |
|  | **Vegetable-derived nitrate intake quintiles** | | | | | |
|  | **Q1**  **n = 1 327** | **Q2**  **n = 1 238** | **Q3**  **n = 1 232** | **Q4**  **n = 1 207** | **Q5**  **n = 1 151** | **P for trend** |
| Systolic blood pressure | ref. | -1.47 (-3.01, 0.07) | -1.68 (-3.23, -0.12) | -1.90 (-3.49, -0.32) | -2.20 (-3.81, -0.58) | 0.024 |
| Diastolic blood pressure | ref. | -0.59 (-1.35, 0.16) | -0.71 (-1.47, 0.05) | -0.69 (-1.46, 0.09) | -1.04 (-1.83, -0.25) | 0.027 |
| Results are analysed by linear regression and are adjusted for age, sex, BMI, smoking status (current/former/never), physical activity (total daily metabolic equivalent), pure alcohol intake (g/d), social economic status (income), marital status, hypercholesterolemia (yes/no), education, and prevalent disease (diabetes, chronic obstructive pulmonary disease, chronic kidney disease, and cancer; entered into the model separately). Results are presented as linear coefficient (95% CI) | | | | | | |

| **Supplemental Table 4. Association between vegetable nitrate intake (mg/d) and blood pressure in people not on antihypertensive medication** | | | | | | |
| --- | --- | --- | --- | --- | --- | --- |
|  | **Vegetable-derived nitrate intake quintiles** | | | | | |
|  | **Q1**  **n = 9 303** | **Q2**  **n = 9 392** | **Q3**  **n = 9 398** | **Q4**  **n = 9 423** | **Q5**  **n = 9 479** | **P for trend** |
| Systolic blood pressure | ref. | -0.95 (-1.48, -0.42) | -1.82 (-2.35, -1.29) | -2.01 (-2.55, -1.47) | -2.49 (-3.04, -1.95) | <0.001 |
| Diastolic blood pressure | ref. | -0.62 (-0.90, -0.34) | -0.92 (-1.20, -0.64) | -1.09 (-1.38, -0.81) | -1.36 (-1.64, -1.07) | <0.001 |
| Results are analysed by linear regression and are adjusted for age, sex, BMI, smoking status (current/former/never), physical activity (total daily metabolic equivalent), pure alcohol intake (g/d), social economic status (income), marital status, hypercholesterolemia (yes/no), education, and prevalent disease (diabetes, chronic obstructive pulmonary disease, chronic kidney disease, and cancer; entered into the model separately). Results are presented as linear coefficient (95% CI). | | | | | | |

| **Supplemental Table 5. Association between non-vegetable nitrate intake (mg/d) and blood pressure** | | | | | | |
| --- | --- | --- | --- | --- | --- | --- |
|  | **Non-vegetable-derived nitrate intake quintiles** | | | | | |
|  | **Q1**  **n = 10 630** | **Q2**  **n = 10 630** | **Q3**  **n = 10 630** | **Q4**  **n = 10 630** | **Q5**  **n = 10 630** | **P for trend** |
| Systolic blood pressure | ref. | -0.06 (-0.058, 0.45) | -0.41 (-0.93, 0.11) | -0.54 (-1.10, -0.02) | -0.05 (-0.47, 0.58) | 0.892 |
| Diastolic blood pressure | ref. | -0.30 (-0.57, -0.04) | -0.58 (-0.85, -0.31) | -0.57 (-0.84, -0.29) | -0.20 (-0.48, 0.07) | 0.183 |
| Results are analysed by linear regression and are adjusted for age, sex, BMI, smoking status (current/former/never), physical activity (total daily metabolic equivalent), pure alcohol intake (g/d), social economic status (income), marital status, hypercholesterolemia (yes/no), education, and prevalent disease (diabetes, chronic obstructive pulmonary disease, chronic kidney disease, and cancer; entered into the model separately). Results are presented as linear coefficient (95% CI). | | | | | | |

| **Supplemental Table 6. 20-year predicted risk of incident CVD and CVD subtypes for males** | | | |
| --- | --- | --- | --- |
|  | **Vegetable nitrate intake** | | **Risk difference**  **(%)** |
|  | **Q1**  **Risk (95% CI)** | **Q3**  **Risk (95% CI)** |  |
| Total CVD | 24.58 (23.16 – 26.06) | 21.79 (20.48 – 23.15) | 2.79 |
| IHD | 9.10 (8.20 – 10.09) | 8.45 (7.61 – 9.38) | 0.65 |
| Ischaemic stroke | 5.33 (4.62 – 6.13) | 4.25 (3.67 – 4.92) | 1.08 |
| Haemorrhagic stroke | 1.12 (0.83 – 1.51) | 1.06 (0.79 – 1.42) | 0.06 |
| PAD | 1.68 (1.37 – 2.06) | 1.39 (1.13 – 1.71) | 0.29 |
| Heart Failure | 3.66 (3.16 – 4.23) | 3.21 (2.76 – 3.73) | 0.45 |
| AF | 11.19 (10.19 – 12.27) | 10.71 (9.75 – 11.74) | 0.48 |
| The 20-year predicted risks (%) of incident CVD and CVD subtypes calculated from logistic regression models. These estimates are for a non-smoking male participant, aged 56 years, with a BMI of 25.5 kg/m^2^, a total daily metabolic equivalent score of 56, with a mean household income of 394 701 – 570 930 DKK/year, and an alcohol intake of 13 g/day. | | | |

| **Supplemental Table 7. 20-year predicted risk of incident CVD and CVD subtypes for females** | | | |
| --- | --- | --- | --- |
|  | **Vegetable nitrate intake** | | **Risk difference**  **(%)** |
|  | **Q1**  **Risk (95% CI)** | **Q3**  **Risk (95% CI)** |  |
| Total CVD | 14.82 (13.90 – 15.79) | 12.94 (12.12 – 13.81) | 1.88 |
| IHD | 4.09 (3.67 – 4.56) | 3.78 (3.39 – 4.22) | 0.31 |
| Ischaemic stroke | 3.77 (3.28 – 4.32) | 2.99 (2.60 – 3.45) | 0.78 |
| Haemorrhagic stroke | 0.80 (0.60 – 1.06) | 0.75 (0.56 – 0.99) | 0.05 |
| PAD | 0.98 (0.80 – 1.19) | 0.80 (0.66 – 0.99) | 0.18 |
| Heart Failure | 2.10 (1.81 – 2.43) | 1.84 (1.58 – 2.13) | 0.26 |
| AF | 7.09 (6.46 – 7.78) | 6.77 (6.17 – 7.43) | 0.32 |
| The 20-year predicted risks (%) of incident CVD and CVD subtypes calculated from logistic regression models. These estimates are for a non-smoking female participant, aged 56 years, with a BMI of 25.5 kg/m^2^, a total daily metabolic equivalent score of 56, with a mean household income of 394 701 – 570 930 DKK/year, and an alcohol intake of 13 g/day. | | | |

**Supplemental Figure 2**


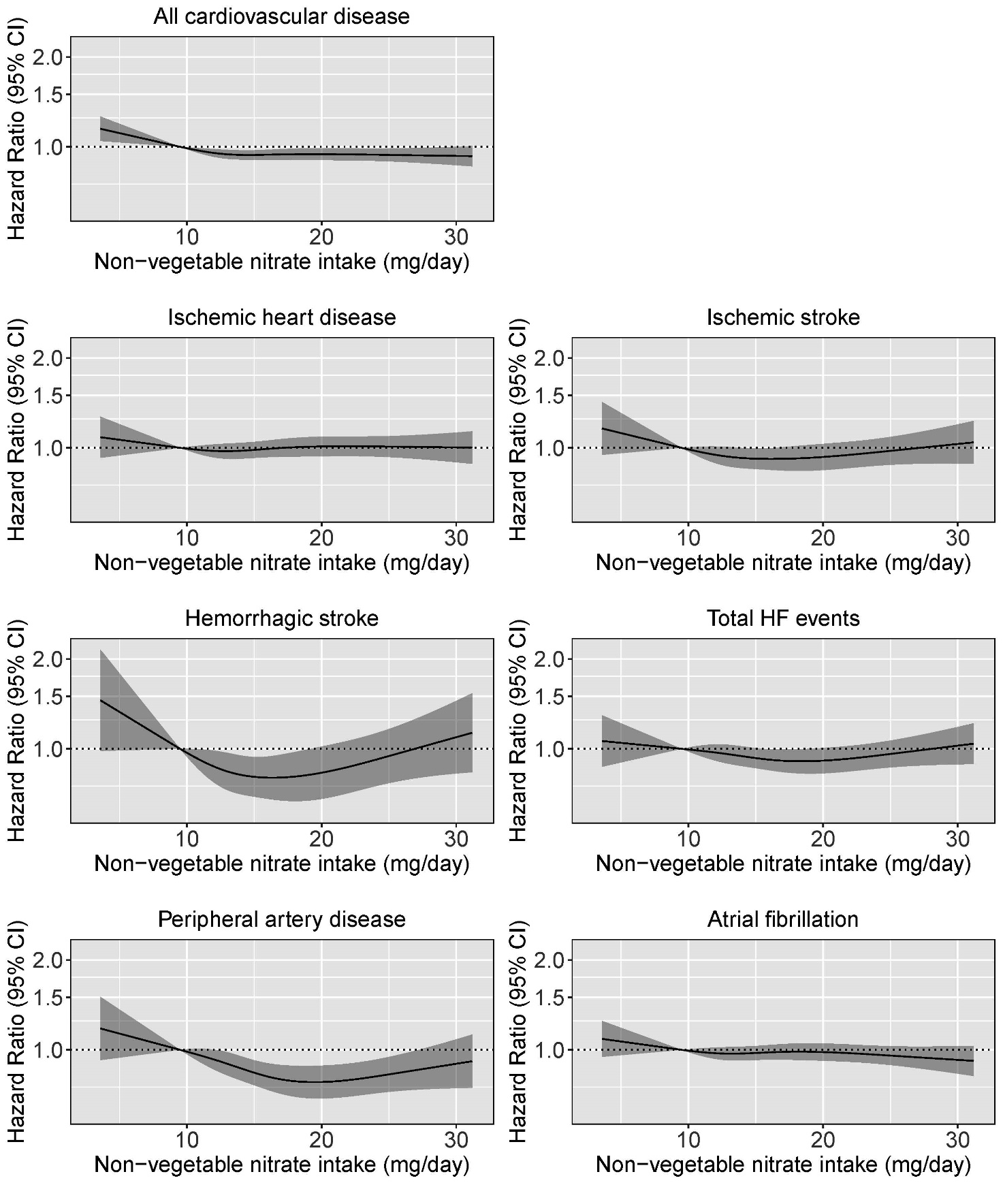

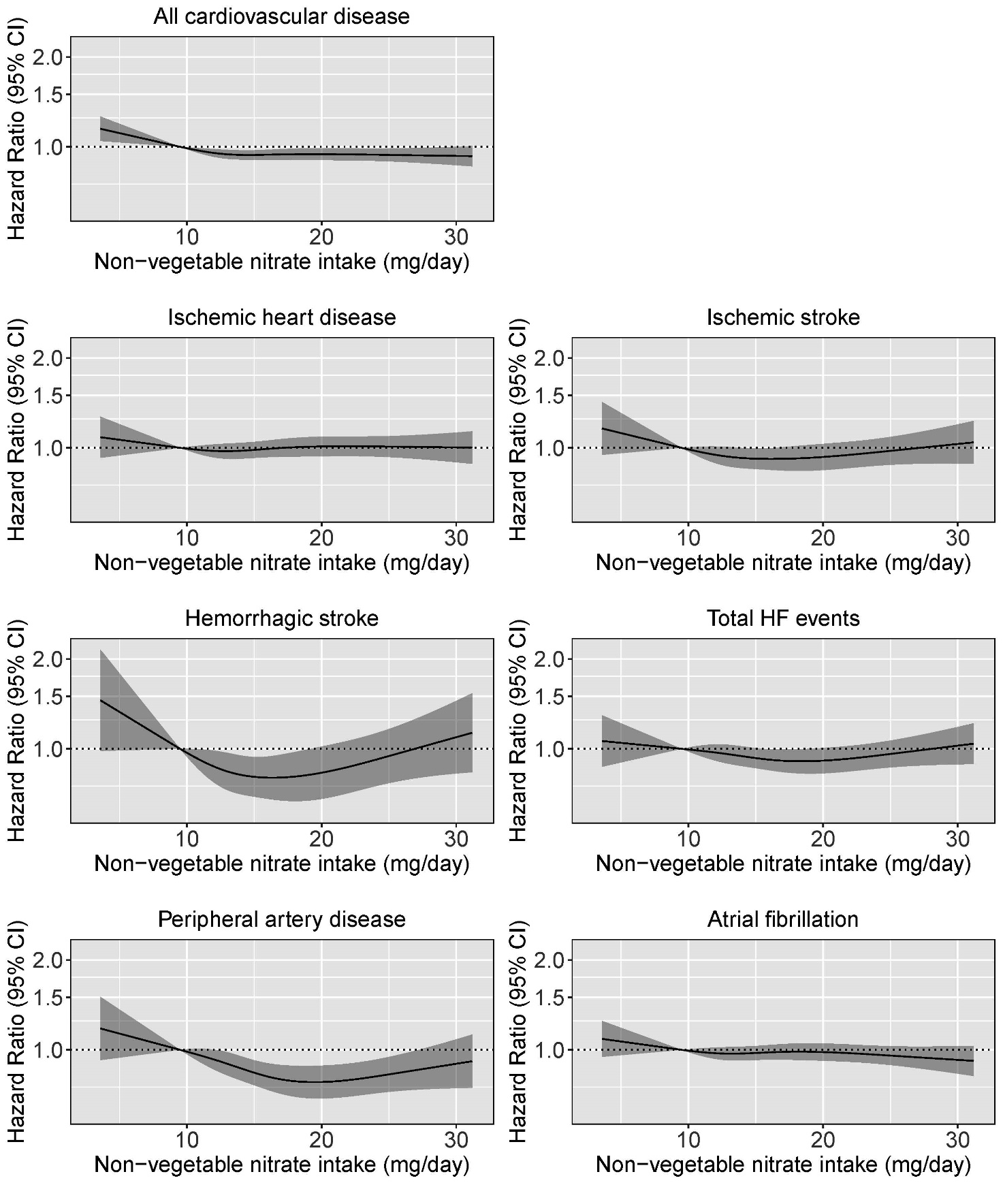

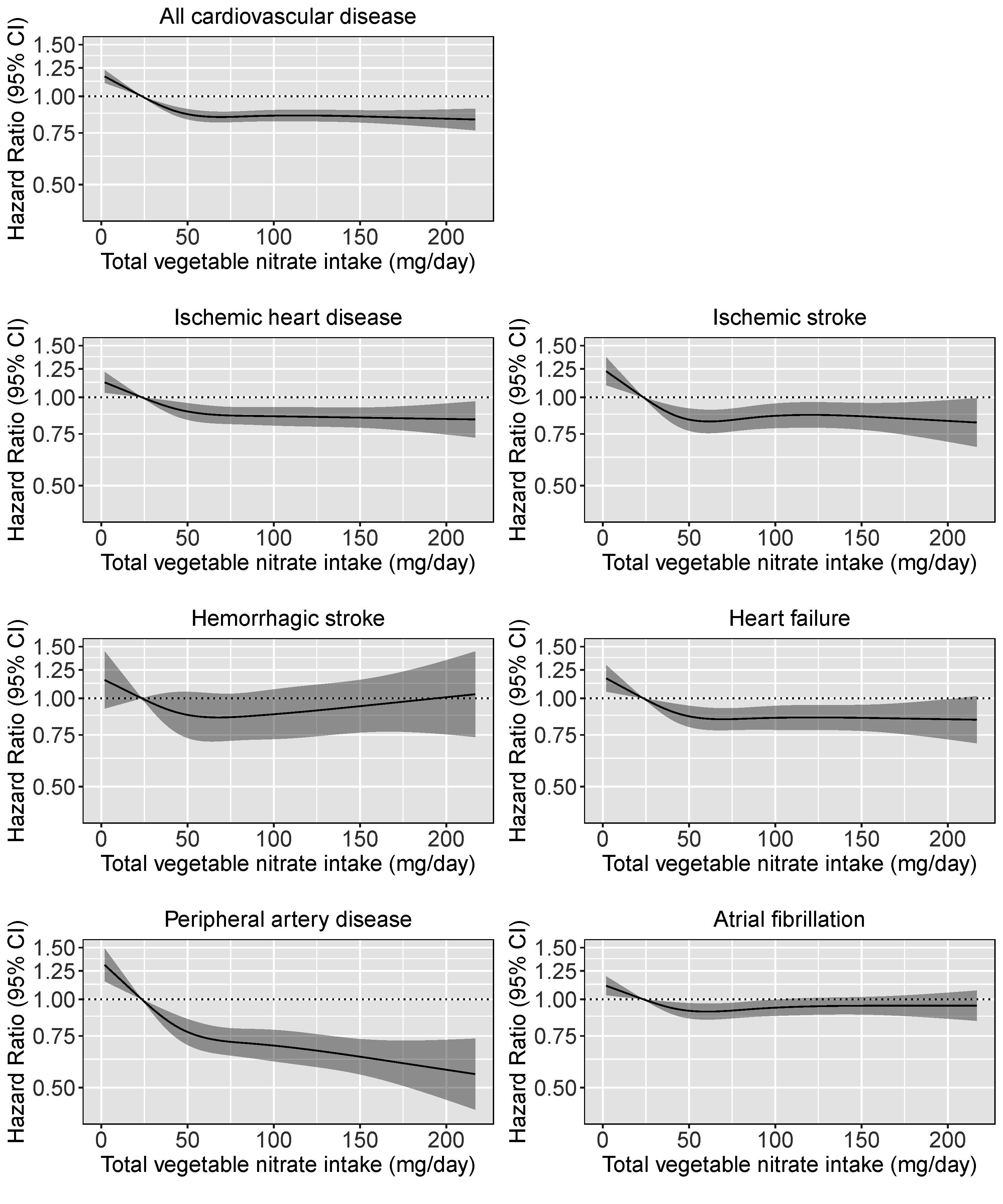


**Supplemental Figure 2.** Cubic spline curves describing the association between non-vegetable nitrate intake and CVD incidence (n =14 088) and the incidence of CVD subtypes; IHD (n = 5 327), ischemic stroke (n = 2 885), hemorrhagic stroke (n = 709), heart failure (n = 3 081), peripheral artery disease (n = 1 867) and atrial fibrillation (n = 6 748). Hazard ratios are based on Cox proportional hazards models adjusted for age, sex, BMI, smoking status, physical activity, alcohol intake, education, social economic status (income), marital status, hypercholesterolemia, diabetes, COPD, chronic kidney disease and cancer and are comparing the specific level of non-vegetable nitrate intake (horizontal axis) to the median intake for participants in the lowest intake quintile (9 mg/day).

| **Supplemental Table 8. Hazard ratios of incident CVD and CVD subtypes by quintiles of non-vegetable nitrate intake** | | | | | |
| --- | --- | --- | --- | --- | --- |
|  | **Non-vegetable nitrate intake quintiles** | | | | |
|  | **Q1**  **(n=10 630)** | **Q2**  **(n=10 630)** | **Q3**  **(n=10 630)** | **Q4**  **(n=10 630)** | **Q5**  **(n=10 630)** |
| Intake (mg/d) * | 9 [3, 11] | 12 [11, 13] | 15 [13, 16] | 17 [16, 19] | 22 [19, 59] |
| **Total CVD** | |  |  |  |  |
| No. events | 2 794 | 2 767 | 2 809 | 2 814 | 2 904 |
| HR (95% CI) |  |  |  |  |  |
| Model 1a | ref. | 0.91 (0.88, 0.94) | 0.88 (0.85, 0.92) | 0.89 (0.85, 0.93) | 0.90 (0.86, 0.94) |
| Model 1b | ref. | 0.95 (0.92, 0.98) | 0.94 (0.91, 0.97) | 0.94 (0.90, 0.98) | 0.94 (0.90, 0.99) |
| Model 2 | ref. | 0.95 (0.92, 0.98) | 0.94 (0.90, 0.98) | 0.95 (0.90, 1.00) | 0.95 (0.89, 1.01) |
| Model 3 | ref. | 0.95 (0.92, 0.99) | 0.94 (0.90, 0.99) | 0.94 (0.89, 1.00) | 0.94 (0.87, 1.02) |
| **IHD** | |  |  |  |  |
| No. events | 988 | 991 | 1 056 | 1 056 | 1 146 |
| HR (95% CI) |  |  |  |  |  |
| Model 1a | ref. | 0.92 (0.88, 0.97) | 0.91 (0.85, 0.97) | 0.93 (0.86, 0.99) | 0.95 (0.88, 1.02) |
| Model 1b | ref. | 0.98 (0.93, 1.03) | 0.98 (0.92, 1.04) | 1.00 (0.93, 1.07) | 1.01 (0.94, 1.09) |
| Model 2 | ref. | 1.00 (0.94, 1.05) | 1.02 (0.95, 1.09) | 1.06 (0.98, 1.15) | 1.10 (0.99, 1.21) |
| Model 3 | ref. | 0.99 (0.93, 1.04) | 1.00 (0.92, 1.08) | 1.02 (0.93, 1.13) | 1.04 (0.91, 1.19) |
| **Ischemic stroke** | |  |  |  |  |
| No. events | 587 | 594 | 556 | 522 | 626 |
| HR (95% CI) |  |  |  |  |  |
| Model 1a | ref. | 0.90 (0.85, 0.97) | 0.87 (0.80, 0.94) | 0.87 (0.79, 0.95) | 0.90 (0.81, 0.99) |
| Model 1b | ref. | 0.94 (0.88, 1.01) | 0.92 (0.85, 1.00) | 0.92 (0.84, 1.01) | 0.95 (0.86, 1.05) |
| Model 2 | ref. | 0.96 (0.90, 1.04) | 0.96 (0.87, 1.05) | 0.97 (0.87, 1.08) | 1.02 (0.90, 1.17) |
| Model 3 | ref. | 0.95 (0.88, 1.03) | 0.94 (0.85, 1.03) | 0.94 (0.82, 1.07) | 0.98 (0.82, 1.17) |
| **Haemorrhagic stroke** | |  |  |  |  |
| No. events | 154 | 143 | 135 | 127 | 150 |
| HR (95% CI) |  |  |  |  |  |
| Model 1a | ref. | 0.83 (0.73, 0.95) | 0.77 (0.67, 0.90) | 0.76 (0.64, 0.91) | 0.84 (0.69, 1.03) |
| Model 1b | ref. | 0.86 (0.76, 0.98) | 0.81 (0.70, 0.94) | 0.80 (0.67, 0.96) | 0.88 (0.72, 1.07) |
| Model 2 | ref. | 0.83 (0.72, 0.95) | 0.75 (0.63, 0.90) | 0.72 (0.58, 0.90) | 0.76 (0.58, 0.99) |
| Model 3 | ref. | 0.86 (0.74, 0.99) | 0.80 (0.66, 0.97) | 0.79 (0.60, 1.03) | 0.85 (0.60, 1.21) |
| **Heart Failure** |  |  |  |  |  |
| No. events | 639 | 597 | 634 | 573 | 638 |
| HR (95% CI) |  |  |  |  |  |
| Model 1a | ref. | 0.90 (0.84, 0.96) | 0.85 (0.79, 0.92) | 0.83 (0.76, 0.90) | 0.86 (0.78, 0.94) |
| Model 1b | ref. | 0.97 (0.91, 1.03) | 0.94 (0.87, 1.01) | 0.91 (0.83, 1.00) | 0.93 (0.84, 1.03) |
| Model 2 | ref. | 0.96 (0.90, 1.03) | 0.92 (0.84, 1.01) | 0.89 (0.80, 0.99) | 0.90 (0.79, 1.02) |
| Model 3 | ref. | 0.93 (0.86, 1.00) | 0.86 (0.78, 0.95) | 0.80 (0.70, 0.91) | 0.76 (0.64, 0.91) |
| **PAD** |  |  |  |  |  |
| No. events | 422 | 370 | 369 | 330 | 351 |
| HR (95% CI) |  |  |  |  |  |
| Model 1a | ref. | 0.84 (0.77, 0.91) | 0.75 (0.68, 0.82) | 0.69 (0.61, 0.77) | 0.69 (0.61, 0.78) |
| Model 1b | ref. | 0.92 (0.85, 1.00) | 0.85 (0.78, 0.94) | 0.79 (0.71, 0.89) | 0.79 (0.70, 0.90) |
| Model 2 | ref. | 0.93 (0.85, 1.01) | 0.87 (0.78, 0.97) | 0.81 (0.71, 0.93) | 0.82 (0.69, 0.97) |
| Model 3 | ref. | 0.93 (0.85, 1.01) | 0.86 (0.76, 0.97) | 0.80 (0.68, 0.94) | 0.80 (0.64, 1.00) |
| **AF** |  |  |  |  |  |
| No. events | 1 306 | 1 320 | 1 342 | 1 402 | 1 378 |
| HR (95% CI) |  |  |  |  |  |
| Model 1a | ref. | 0.95 (0.91, 0.99) | 0.94 (0.90, 1.00) | 0.96 (0.90, 1.02) | 0.96 (0.90, 1.02) |
| Model 1b | ref. | 0.97 (0.93, 1.02) | 0.97 (0.92, 1.03) | 0.98 (0.93, 1.05) | 0.97 (0.91, 1.04) |
| Model 2 | ref. | 0.96 (0.92, 1.01) | 0.95 (0.90, 1.01) | 0.96 (0.89, 1.03) | 0.93 (0.86, 1.02) |
| Model 3 | ref. | 0.97 (0.92, 1.02) | 0.96 (0.90, 1.03) | 0.97 (0.89, 1.06) | 0.95 (0.84, 1.07) |
| Hazard ratios (95% CI) for incident CVD and CVD subtypes during 23 years of follow, up, obtained from restricted cubic splines based on Cox proportional hazards models. Model 1 adjusted for age and sex; Model 1b adjusted for all covariates in Model 1 plus BMI, smoking status (current/former/never), physical activity (total daily metabolic equivalent), pure alcohol intake (g/d), social economic status (income), marital status, hypercholesterolemia (yes/no), education, and prevalent disease (diabetes, chronic obstructive pulmonary disease, chronic kidney disease, and cancer; entered into the model separately); Model 2 adjusted for all covariates in Model 1b plus energy; Model 3: adjusted for all covariates in Model 2 plus intakes (g/d) of fish, red meat, polyunsaturated fatty acids, monounsaturated fatty acids, saturated fatty acids, and all fruit.  *Median; range in parentheses (all such values). | | | | | |

**
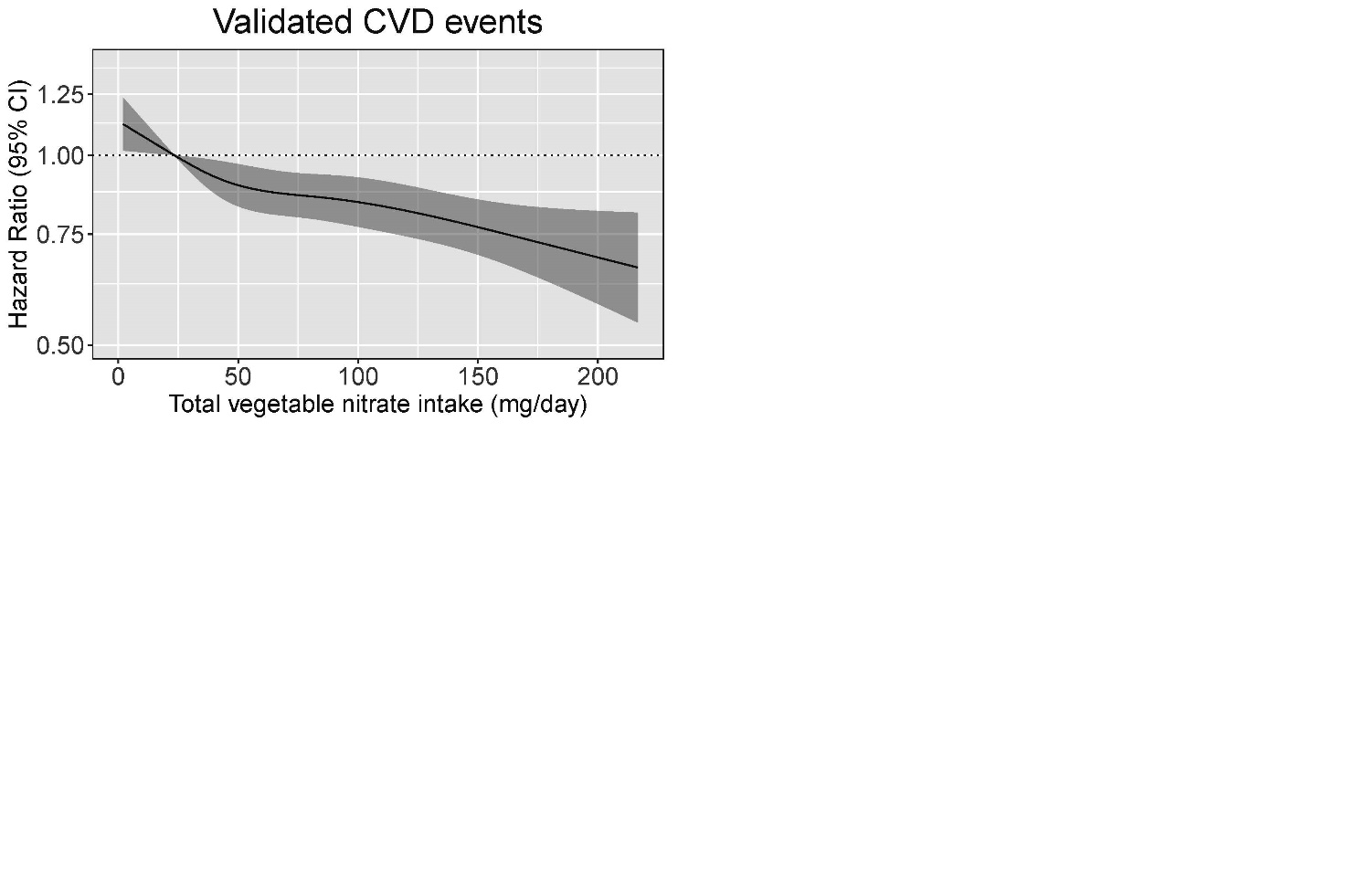
 Supplemental Figure 3**

**Supplemental Figure 3.** Cubic spline curves describing the association between vegetable nitrate intake (mg/day) and validated cases of CVD (n = 3,430). Hazard ratios are based on Cox proportional hazards models adjusted for age, sex, BMI, smoking status, physical activity, alcohol intake, education, social economic status (income), marital status, hypercholesterolemia, diabetes, COPD, chronic kidney disease and cancer and are comparing the specific level of vegetable nitrate intake (horizontal axis) to the median intake for participants in the lowest intake quintile (23 mg/day).

| **Supplemental Table 9. Association between incident CVD and vegetable nitrate intake stratified by tertiles of total vegetable intake** | | | | | |
| --- | --- | --- | --- | --- | --- |
|  | **Vegetable nitrate intake quintiles** | | | | |
| **Baseline vegetable intake tertile** | **Q1**  **(n=10 630)** | **Q2**  **(n=10 630)** | **Q3**  **(n=10 630)** | **Q4**  **(n=10 630)** | **Q5**  **(n=10 630)** |
| **1** n (events) | 2 967 | 1 762 | 462 | 189 | 6 |
| HR (95% CI) | ref. | 0.90 (0.84, 0.95) | 0.86 (0.79, 0.94) | 0.93 (0.81, 1.07) | 1.05 (0.76, 1.45) |
| **2** n (events) | 312 | 1 080 | 1 438 | 1 316 | 354 |
| HR (95% CI) | ref. | 0.90 (0.83, 0.97) | 0.83 (0.73, 0.94) | 0.83 (0.74, 0.93) | 0.79 (0.67, 0.96) |
| **3** n (events) | 30 | 195 | 825 | 951 | 2 201 |
| HR (95% CI) | ref. | 0.95 (0.91, 0.99) | 0.89 (0.80, 0.99) | 0.84 (0.71, 0.99) | 0.91 (0.78, 1.05) |
| Hazard ratios (95% CI) for incident CVD during 23 years of follow, up, obtained from Cox proportional hazards models using model 1b for adjustment: age, sex, BMI, smoking status (current/former/never), physical activity (total daily metabolic equivalent), pure alcohol intake (g/d), social economic status (income), marital status, hypercholesterolemia (yes/no), education, and prevalent disease (diabetes, chronic obstructive pulmonary disease, chronic kidney disease, and cancer; entered into the model separately) | | | | | |

**References**

1. Hansen CP, Overvad K, Tetens I, Tjønneland A, Parner ET, Jakobsen MU, Dahm CC. Adherence to the Danish food-based dietary guidelines and risk of myocardial infarction: a cohort study. Public Health Nutr 2018;21(7):1286-96.

2. Lasota AN, Overvad K, Eriksen HH, Tjønneland A, Schmidt EB, Grønholdt ML. Validity of peripheral arterial disease diagnoses in the Danish National Patient Registry. Eur J Vasc Endovasc Surg 2017;53(5):679-85.

3. Lühdorf P, Overvad K, Schmidt EB, Johnsen SP, Bach FW. Predictive value of stroke discharge diagnoses in the Danish National Patient Register. Scand J Public Health 2017;45(6):630-6.
